# Identifying Financially Sustainable Pricing Interventions to Promote Healthier Beverage Purchases in Small Neighborhood Stores

**DOI:** 10.5888/pcd15.160611

**Published:** 2018-01-25

**Authors:** Claudia Nau, Shiriki Kumanyika, Joel Gittelsohn, Atif Adam, Michelle S. Wong, Yeeli Mui, Bruce Y. Lee

**Affiliations:** 1 Kaiser Permanente Southern California, Department of Research and Evaluation, Pasadena, California; 2 Department of Biostatistics and Epidemiology, University of Pennsylvania Perelman School of Medicine, Philadelphia, Pennsylvania; 3 Global Obesity Prevention Center, Johns Hopkins University, Baltimore, Maryland; 4 Center for Human Nutrition, Department of International Health, Johns Hopkins Bloomberg School of Public Health, Baltimore, Maryland; 5 Department of Health Policy and Management, Johns Hopkins Bloomberg School of Public Health, Baltimore, Maryland; 6 Food Systems Planning and Healthy Communities Lab, University at Buffalo, State University of New York, Buffalo, New York; 7 Carey Business School, Johns Hopkins University, Baltimore, Maryland

## Abstract

**Introduction:**

Residents of low-income communities often purchase sugar-sweetened beverages (SSBs) at small, neighborhood “corner” stores. Lowering water prices and increasing SSB prices are potentially complementary public health strategies to promote more healthful beverage purchasing patterns in these stores. Sustainability, however, depends on financial feasibility. Because in-store pricing experiments are complex and require retailers to take business risks, we used a simulation approach to identify profitable pricing combinations for corner stores.

**Methods:**

The analytic approach was based on inventory models, which are suitable for modeling business operations. We used discrete-event simulation to build inventory models that use data representing beverage inventory, wholesale costs, changes in retail prices, and consumer demand for 2 corner stores in Baltimore, Maryland. Model outputs yielded ranges for water and SSB prices that increased water demand without loss of profit from combined water and SSB sales.

**Results:**

A 20% SSB price increase allowed lowering water prices by up to 20% while maintaining profit and increased water demand by 9% and 14%, for stores selling SSBs in 12-oz cans and 16- to 20-oz bottles, respectively. Without changing water prices, profits could increase by 4% and 6%, respectively. Sensitivity analysis showed that stores with a higher volume of SSB sales could reduce water prices the most without loss of profit.

**Conclusion:**

Various combinations of SSB and water prices could encourage water consumption while maintaining or increasing store owners’ profits. This model is a first step in designing and implementing profitable pricing strategies in collaboration with store owners.

## Introduction

Researchers have pointed to the lack of obesity prevention strategies that consider retailer revenue and profit (1,2). Water consumption has been promoted to reduce calorie intake otherwise obtained from sugar-sweetened beverages (SSBs) (3–5) and to provide a range of other health benefits (5,6). Consumer demand for beverages is sensitive to price changes, and some obesity prevention interventions have lowered the price of bottled water to improve beverage consumption (7,8). However, lowering prices may negatively affect retailer profit if not compensated (9). Locally owned retail stores, in particular, operate under small profit margins and have limited resources and motivation to implement and maintain pricing interventions that may threaten their bottom line (10).

We assessed feasibility of coordinated price changes of bottled water and SSBs as a profitable public health strategy in small stores in low-income urban neighborhoods in Baltimore, Maryland. These small, privately owned stores — referred to here as “corner stores” because they are often situated at corner locations — play an important role as beverage providers in many low-income urban neighborhoods that do not have access to larger retail food stores (10,11). SSBs are among the best-selling products in these stores, constituting a significant part of the stores’ revenue (7,12).

To design a financially self-sustaining pricing intervention, we built simulation models that mimicked the day-to-day stocking and sales of SSBs and bottled water in 2 Baltimore corner stores. These 2 stores served as case studies to assess feasibility of our strategy. An explicit dual focus on public health goals and retailer profit allowed us to design an intervention that would be beneficial for community health and for retailers. Using simulation models allowed us to estimate sales and profit under different pricing scenarios without disrupting stores’ operations. We show how results from our models can serve as a first step toward implementing profitable pricing interventions in collaboration with store owners.

## Methods

### Theoretical framework and approach

Our theoretical framework and modeling approach stem from inventory control theory, which is concerned with optimizing supply and retail processes (13). Inventory control theory focuses on factors such as wholesale prices, storage, and sales prices that regulate stocking and sale of a store’s inventory with the goal of maximizing sales and profits (13).We used discrete-event simulation (DES), an approach frequently used in inventory control research, to build inventory models that model the daily product flow of 2 beverage categories, bottled water and SSBs (13,14).

### Inventory model

To identify pricing combinations that increase both demand for and profitability of healthier beverages (14), we built 2 inventory models that represented the beverage inventory and sales of 2 actual corner stores (store A and store B), in different low-income neighborhoods in Baltimore. Store A and store B were typical examples of Baltimore corner stores and were chosen because we were provided with information on their beverage sales by a prior survey they had participated in. They were comparable in size, and each had beverage coolers installed along the length of one of the side walls. We incorporated the average sales price of SSBs and 16-oz bottles of water sold in each store in the model as well as wholesaler costs. Each model also represented the demand for beverages of the community members living around both stores. Store A sold SSBs predominantly in 16- to 20-oz bottles (out of 5 coolers, only half a cooler was stocked with SSB cans); store B sold SSBs exclusively in 12-oz cans. Stores A and B thus serve as case studies for different inventory profiles. Each inventory profile was modeled separately, because sales prices of SSB bottles and cans were different, and thus, the same percentage change in sales prices yielded different absolute changes in sales prices, demand, and profit across stores. As we show below, the inventory of both SSBs and water was large compared with the demand, and store owners restocked products most days (10); therefore, running out of stock was not an issue. To calculate daily profit for changing prices, we assumed that restocking occurred the same day sales were made.

By using daily demand, average prices, and wholesaler costs of SSBs and water stocked in each store, we calculated baseline profit under current prices. We then used price elasticities, explained in detail below, to simulate demand and profit in each store when water prices decreased by $0.01 increments and SSB prices increased by $0.01 increments until SSB prices had increased by 20% and water prices decreased by 20%. We assessed all possible price combinations of SSBs and water, paying particular attention to the following 3 price combinations: 1) the price combination that maximized the demand for water while maintaining or increasing profit (the ideal price point from a public health point of view), 2) the price combination that maximized profit while at least maintaining current demand for water (the ideal price point from the store owner’s perspective), and 3) the pricing combination that produced an equal percentage increase of water demand and profit (as an example of a compromise price point for both retail and public health stakeholders).

### Data sources

We collected detailed information on the beverage inventory, sales prices, and sales from store A and store B, including the number of items and the sales price of each SSB brand. Beverage wholesale costs were obtained by directly recording brand-specific prices from the 2 principal suppliers of Baltimore corner stores. Different prices for the same beverage were averaged between wholesalers. We only considered sodas and fruit drinks. We calculated an average wholesale cost and an average sales price for SSB bottles and cans and for water for each store. The average price was weighted by the number of bottles or cans of each specific beverage stocked and sold in each store.

We used store owner recall of sales for each beverage category to measure demand. Data from stores A and B were collected as part of a prior study (15).

To model the response of demand to price changes, we drew estimates of the effects of price changes on demand for SSBs and water in low-income populations — so-called own-price-elasticities — from a study by Lin et al (16). Lin et al used nationally representative data and found that in low-income populations, a 1% increase in water prices caused a 0.95% decrease in demand. Similarly, a 1% increase in SSB prices decreased demand by 0.72%. Lin et al found that water prices do not affect SSB demand and vice versa (16). In our data set, a 20% increase amounted to a maximum price of $1.26 for SSB bottles and $0.93 for cans. Analogously, for water, a price decrease of 20% lead to a sales price of $0.80 per 16-oz bottle. We limited price changes to a maximum of 20% because Lin et al found that demand within this range could be approximated by their estimates (B-H. Lin, email communication, September 9, 2014).

### Model inputs

The inventory was set to 544 SSB bottles in model A (representing store A) and to 588 SSB cans in model B (representing store B), mirroring the actual number of SSBs in each store (Table 1). Starting values for sales prices and wholesale costs were set to the average sales price and wholesale cost calculated for SSB bottles in store A ($1.05 and $0.50, respectively) and SSB cans in store B ($0.77 and $0.36, respectively). In both stores, the inventory, price, and sales of 16-oz bottles of water were nearly identical and were set at 41 bottles in stock with a sales price of $1.00 and a per-item wholesale cost of $0.12 for both modeling scenarios. Baseline levels of daily demand for SSBs varied between stores (24 bottles for store A and 20 cans for store B); sales of bottled water were comparable and were set to be 15 per day for both stores. We calculated the current profit generated when prices were set to the current SSB and water sales prices by multiplying the number of sales with the average price and subtracting the wholesaler costs. Profit in model A was $26.38 and in model B was $21.53. Profit from any pricing intervention had to be equal to or higher than the current profit of each store. The discrete event simulation model was programmed in R 3.2.2 (R Foundation for Statistical Computing).

**Table 1:** Number Stocked and Sold and Average Price and Wholesale Cost for Sugar-Sweetened Beverages (SSBs) (12-oz cans and 16- to 20-oz bottles), and Water (16-oz) in 2 Baltimore, Maryland, Corner Stores, 2014, and Inputs for Simulation Scenario for Each Store

Product	Inventory		Simulation	
	Store A, SSB Bottles	Store B, SSB Cans	Scenario A, SSB Bottles and Water Bottles	Scenario B, SSB Cans and Water Bottles
**SSB bottles**				
No. of SSB bottles stocked	544	NA	544	NA
Average sales price of SSB per bottle^a^, $	1.05		1.05	
Average cost at wholesaler^b^, $	0.50		0.50	
Average SSB sales per day^c^, bottles	24		24	
**SSB cans**				
No. of SSB cans stocked	NA	588	NA	588
Average sales price of SSB per can^a^, $		0.77		0.77
Average cost at wholesaler^b^, $		0.36		0.36
Average SSB sales per day^c^, cans		20		20
**Water bottles**				
No. of water bottles stocked	40	42	41	41
Average sales price of water per bottle, $	1.00	1.00	1.00	1.00
Average cost at wholesaler, $	0.12	0.12	0.12	0.12
Average water sales per day, bottles	15	14	15	15
**Total daily profit at baseline prices**	NA	NA	26.38	21.53

Abbreviation: NA, not applicable.

a Average sale price of SSB bottles and cans are the quantity-weighted average sale prices of specific beverages sold in each store. The data were collected by the authors.

b Data on average sales per day come from questionnaires of the B’More Healthy Communities for Kids study (15).

c In store A, out of 5 coolers with SSBs, less than half a cooler was stocked with SSB cans. Store B was carrying SSB cans exclusively. Therefore, the simplifying assumption is made that Store A stocked only SSB bottles.

## Results

### Price combinations of SSB bottles and water (model A)

**Figure 1:**
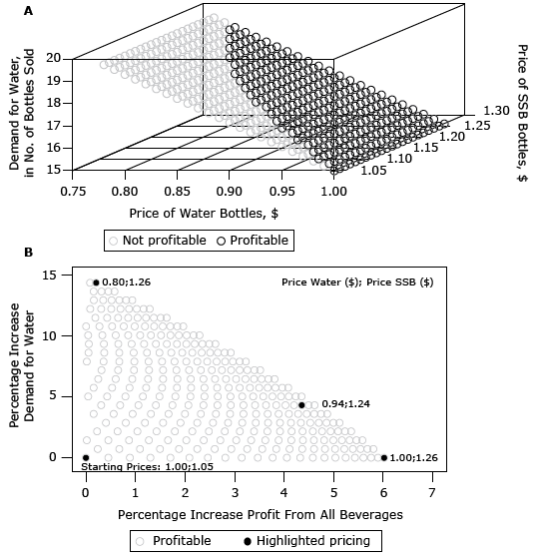
Water demand and profit for coordinated price changes for selling bottles of sugar-sweetened beverages (SSBs) for corner store A, Baltimore, Maryland, 2014. Panel A shows absolute demand of water over prices of water and bottles of SSBs. Panel B shows only profitable price combinations for percentage change of water demand in relation to profit change.

Panel A in Figure 1 plots the daily demand of water bottles against all price combinations of water and SSB bottles. Our results showed that water demand increased as water prices decreased. However, SSB bottle prices had to increase simultaneously, or profit declined. Coordinated price changes of water and SSB bottles allowed water demand to increase from 15 to a maximum of 17.15 bottles per day at a sales price of $0.80.

Figure 1, panel B, uses all price combinations within the profitable area of Figure 1, panel A (black symbols). For each price combination, we plotted the percentage increase in water demand against the percentage increase in profit that occurred at that price combination (Figure 1, panel B). We found that a price combination of $0.80 (20% decrease) for water and $1.26 for SSB bottles generated a maximum increase of 14.36% in water purchases (2.15 bottles per day) while maintaining the same level of profit as current prices. Maximum total profits are achieved when water prices remain at $1.00 and SSB prices are set to the maximum sales price of $1.26, resulting in a 6.00% profit increase. Equal percentage increases of profit (4.36%) and water demand (4.31%) are achieved at a price combination of $0.94 and $1.24 for water and SSB bottles, respectively.

### Price combinations of SSB cans and water (model B)

**Figure 2:**
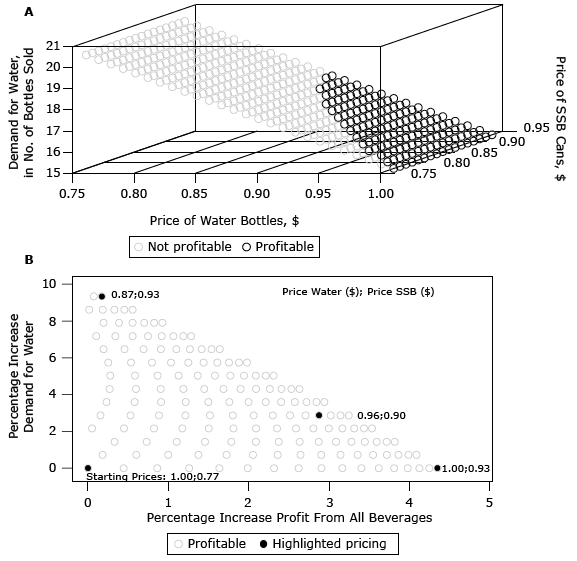
Water demand and profit for coordinated price changes for selling cans of sugar-sweetened beverages (SSBs) for corner store B, Baltimore, Maryland, 2014. Panel A shows absolute demand of water over prices of water and cans of SSBs. Panel B shows only profitable price combinations for percentage change of water demand in relation to profit change.

Figure 2 represents results for store B selling SSB cans analogously to Figure 1. There are fewer profitable price combinations for Store B, as indicated by the smaller area of black symbols in Figure 2, panel A. Panel B of Figure 2 further shows that the maximum increase in water demand is smaller in store B (9.33%) compared with store A (14.36%). Prices that maximize water demand are $0.87 for water and $0.93 for SSB. Similarly, the maximum profit increase in store B was 4.25%, at prices of $1.00 for water and $0.93 for SSB cans, which is less than in store A (6.00%). Equal increases in demand and profit in store B were achieved at 2.87% when water and SSBs were priced $0.96 and $0.90, respectively.

### Sensitivity analyses

The relative demand of water and SSBs at baseline (before any price change) determines how much the price of water can be lowered for a given increase in SSB price while maintaining profit. Therefore, the potential maximum improvement of profit and water demand also varies with the relative difference in the baseline demand of SSBs and water. To understand whether there are baseline conditions that do not allow improving water demand or profit, we used store B’s data to recalculate price combinations and maximum improvement of profit and demand under the assumption that, all else being equal, the demand of SSB cans was first increased by 1 unit increments from current levels to 10 times the initial demand. Then, keeping SSB demand constant at current levels, we allowed baseline demand of water to increase until demand was multiplied by a factor of 10. We chose store B because soda cans provide a smaller profit margin; therefore, they provide less buffer to counter-finance changes in water prices. As before, SSB prices were increased by no more than 20%.

In the current scenario, store B sold 1.3 SSB cans for each water bottle per day. Results from our sensitivity analysis (Table 2) show that doubling baseline SSB demand would increase the maximally attainable profit compared with current demand levels (6.13% vs 4.25%). The maximum attainable demand would increase from 9.33% to 15.80% at a sales price of $0.78 for water and $0.93 for SSBs.

**Table 2:** Sensitivity Analysis Showing the Potential for Improving Demand and Profit at Varying Starting Sales Volumes of Water and Sugar-Sweetened Beverage (SSB) Cans for Corner Store B in Baltimore, Maryland, 2014

Analysis	Price of Water, $	Price of SSB, $	Percentage Improvement in Profit	Percentage Improvement in Water Demand
**Current demand store B: SSB cans sold = 20, water bottles = 15**				
Maximum increase in profit	1.00	0.93	4.25	0.00
Maximum increase in demand	0.87	0.93	0.07	9.33
Equal relative improvement of profit and demand	0.96	0.90	2.87	2.87
**Double SSB demand in store B: SSB cans sold = 40, water bottles = 15**				
Maximum increase in profit	1.00	0.93	6.13	0.00
Maximum increase in demand	0.78	0.93	0.14	15.80
Equal relative improvement of profit and demand	0.94	0.89	4.26	4.31
**Double water demand in store B: SSB cans sold = 20, water bottles = 30**				
Maximum increase in profit	1.00	0.93	2.63	0.00
Maximum increase in demand	0.93	0.93	0.03	5.03
Equal relative improvement of profit and demand	0.98	0.87	1.45	1.44

If, all else being equal, the demand of water at baseline were higher than the current demand for water, then, to maintain profit, water prices could not be lowered as much as in the current scenario. If baseline water demand were doubled, profit could still be increased, but would be much less than under the current scenario (2.63% improvement of profit vs 4.25% in the current scenario) and maximum increase in water demand would be 5.03% compared with 9.33% (Table 2). An equal percentage improvement of demand and profit would be reached at approximately 1.45%. Even if the baseline demand for water were 10 times higher than in the original scenario (ie, if the store would sell 150 bottles of water and 20 SSB cans per day) water demand could be increased minimally by 0.72% at price points of $0.99 and $0.84 for water and SSBs, respectively, while profit would remain unchanged (results not shown).

## Discussion

To our knowledge, this is the first public health study to explore a corner store intervention that explicitly considers profitability for retailers. Our simulations allowed us to identify a range of plausible pricing combinations that are likely to improve beverage consumption and profit. We found that a store stocking SSB bottles could increase water demand by up to 14.36% and profit by up to 6.00% through coordinated price changes. A store selling SSB cans, which are cheaper and less profitable than SSB bottles, could increase demand by up to 9.33% and profit by 4.25%. Although the potential for improvement was smaller in the store selling SSB cans, our results indicate that our strategy can be successfully implemented in stores with different inventory profiles. Sensitivity analysis further showed that our pricing strategy was robust to changes in the demand structure of SSBs and water. We also found that our strategy would be most effective in stores where SSB demand and the need to incentivize water consumption are the highest.

Estimates of price elasticities vary slightly across studies. Additional sensitivity analysis assessed whether our coordinated pricing strategy was robust to different price elasticities. By using price elasticities for low-income populations of 2 other studies (17,18), we found that water demand might possibly be increased even more than our initial results suggested. Overall, we found that our pricing strategy worked under alternative demand elasticity scenarios (Appendix).

We demonstrated that coordinated price changes could improve water consumption while maintaining store profit under a wide range of scenarios. Prior research has shown that pricing is an effective tool that may lead not only to ad hoc, short-term changes in consumer behaviors (19) but also to habituation to healthier products over the long run (20). Pricing has been cited as a particularly important factor in purchasing decisions by low-income and African American customers (21,22), who are priority populations for public health interventions to lower SSB consumption (23).

We built a simulation model to identify plausible pricing combinations because conducting pricing experiments in corner stores is challenging. Optimizing a coordinated pricing strategy requires assessment of many different pricing combinations. Although store owners can set their own prices, most do not have digital cash registers that would allow tracking changes in demand in response to price changes (24). More importantly, increasing prices, even after a low-price promotion, causes customers to voice dissatisfaction and store owners to fear losing customers (25). Thus, a simulation model is an undisruptive first step to assess feasibility and eventually inform in-store experiments.

As a next step toward implementing our profitable pricing strategy, we plan to use stakeholder-involved modeling techniques to improve our model. Stakeholder-involved modeling will allow us to add mechanisms that store owners deem important and that are not yet captured in the model (26). Stakeholder-involved modeling has been found to increase model validity and stakeholder buy-in (27,28). We anticipate that issues such as the proximity to competitors are likely of concern for storeowners and need to be integrated into the model. The effect of other marketing and public health intervention tools, such as product placement or caloric information display, may also be incorporated.

Beyond the limitations of our model that can be addressed with stakeholder-involved modeling, there are other limitations that are inherent in our data and approach. For example, daily demand obtained from store owner recall may be subject to recall bias. Our results also depended on price elasticities that were derived from nationally representative data on beverage purchases from low-income customers for home consumption (16). Purchases of SSBs in 16- to 20-oz bottles or 12-oz cans in corner stores are more likely to be for immediate consumption away from home. These purchases might be more or less sensitive to price changes than those for home consumption. Furthermore, Lin et al combine fruit drinks and sodas into a single SSB category. This approach assumes that both beverages have the same price elasticity. Some research found moderate differences between the elasticities of these beverages (17,18).

Our study has an explicit dual focus on community health and retailer profit. Beyond deriving a strategy for self-sustaining promotions for bottled water, we introduce an intervention strategy that can be generalized to other products to ultimately improve the consumption patterns of low-income populations and support small businesses in low-income communities.

## Appendix

This appendix is available for download as a Microsoft Word document.
