# Supplementary material for: Identifying Financially Sustainable Pricing Interventions to Promote Healthier Beverage Purchases in Small Neighborhood Stores
Source: Prev Chronic Dis. 2018 Jan 25;15:E12. doi: 10.5888/pcd15.160611 (PMC5798217; doi:10.5888/pcd15.160611)
Supplement: Supplementary file 1 [file 16_0611_appendix.docx]

Appendix. Additional Price Elasticities Analyses

Price elasticities indicate by what percentage the demand for a product decreases when its price increases by 1% (1). Little research has assessed price elasticity for low-income populations. Depending on the data and method used to derive price elasticities, studies find slightly different estimates. Two studies that assess price elasticities for low-income populations found price elasticities for water to be −0.95 and −0.82 while having near identical results for price elasticities for sugar-sweetened beverages (SSBs) (−0.72 and −0.73) (1,2). These two publications assessed the effect of price changes on ad-hoc demand. In an analysis assessing the long-term change of demand in response to price changes Zhen et al (2010) account for habit formation and estimate long term price elasticity for low-income populations to be −1.22 for SSB and −1.10 for water (3).

In our primary analysis, we use price elasticities from Lin et al (2011) (1). To assess whether our strategy of coordinated price changes of water and SSBs would allow us to increase water demand while maintaining profit under alternative demand elasticity scenarios, we re-ran our analyses with two alternative price elasticities by Zhen et al (2010) and Zhen et al (2014) (2,3).

Table A.1 presents the maximum increases of demand and profit using Lin’s price elasticities and the alternative price elasticity scenarios for store A (selling SSB mostly in 16- to 20-oz bottles). Table A.2 shows the same information for store B (selling exclusively SSBs in cans). In all scenarios, our price strategy would produce the desired results. In fact, both of Zhen’s price elasticities suggest we could increase water consumption more than what price elasticities by Lin showed. Price elasticities by Zhen et al (2014) project a maximum increase in water demand of 24.60% in store A and 16.40% in store B compared to 14.36% and 9.33% when using Lin et al (2011) price elasticities. Increases in long-term demand as estimated with price elasticities by Zhen et al (2010) are 21.09% for store A and 14.30% for store B.

The maximum increase in profit is also higher under the alternative elasticity scenarios. Using Zhen et al (2010) price elasticities for ad-hoc demand we find that profit could be maximally increased by up to 9.09% compared to 6.00% in store A, and by 6.62% compared to 4.25% in store B. Zhen et al (2010) long-term price elasticities suggest that the maximum increase in profit over the long run is however lower than in both ad-hoc scenarios. In the long-term we can expect only maximum increases of 2.58% (store A) and 1.81% in profit (store B) compared to 6.00% and 4.25% under Lin’s price elasticity scenarios for ad-hoc demand.

Overall, we find that our pricing strategy would be at least as or more successful in increasing water demand while maintaining profit under alternative demand elasticity scenarios. We chose to discuss the most conservative estimates in our manuscript.

**References**

1. Lin B-H, Smith TA, Lee J-Y, Hall KD. Measuring weight outcomes for obesity intervention strategies: the case of a sugar-sweetened beverage tax. Econ Hum Biol 2011;9(4):329–41. [PubMed](https://www.ncbi.nlm.nih.gov/entrez/query.fcgi?cmd=Retrieve&db=PubMed&list_uids=21940223&dopt=Abstract) <http://dx.doi.org/10.1016/j.ehb.2011.08.007>
2. Zhen C, Finkelstein EA, Nonnemaker J, Karns S, Todd JE. Predicting the effects of sugar-sweetened beverage taxes on food and beverage demand in a large demand system. Am J Agric Econ 2014;96(1):1–25. [PubMed](https://www.ncbi.nlm.nih.gov/entrez/query.fcgi?cmd=Retrieve&db=PubMed&list_uids=24839299&dopt=Abstract) <http://dx.doi.org/10.1093/ajae/aat049>
3. Zhen C, Wohlgenant M, Karns S, Kaufman P. Habit formation and demand for sugar-sweetened beverages. Am J Agric Econ 2011;93(1):175–93. <http://dx.doi.org/10.1093/ajae/aaq155>

**Table A.1. Maximum Improvement in Profit and Demand Under Alternative Price Elasticity Scenarios for Store A (Selling Sugar-Sweetened Beverages [SSB] in 16- to 18-oz Bottles), Baltimore, Maryland, 2014**

| **Scenario** | **Price Water in $** | **Price SSB in $** | **Percentage Increase Profit** | **Percentage Increase Demand** |
| --- | --- | --- | --- | --- |
| **Lin et al (2011)^a^**  **SSB: -0.72**  **Water: -0.95** |  |  |  |  |
| Max profit | 1.00 | 1.26 | 6.00 | 0.00 |
| Max demand | 0.80 | 1.26 | 0.21 | 14.36 |
| **Zhen et al (2014)^b^**  **SSB** −**0.73**  **Water** −**0.82** |  |  |  |  |
| Max profit | 1.00 | 1.26 | 9.09 | 0.00 |
| Max demand | 0.70 | 1.26 | 0.16 | 24.60 |
| **Zhen et al (2010)^c^**  **Long-term elasticity**  **SSB** −**1.22**  **Water** −**1.10** |  |  |  |  |
| Max profit | 1.00 | 1.21 | 2.58 | 0.00 |
| Max demand | 0.81 | 1.23 | 0.01 | 21.09 |

^a^ Lin B-H, Smith TA, Lee J-Y, Hall KD. Measuring weight outcomes for obesity intervention strategies: the case of a sugar-sweetened beverage tax. Econ Hum Biol 2011;9(4):329–41. [PubMed](https://www.ncbi.nlm.nih.gov/entrez/query.fcgi?cmd=Retrieve&db=PubMed&list_uids=21940223&dopt=Abstract) <http://dx.doi.org/10.1016/j.ehb.2011.08.007>

^b^ Zhen C, Finkelstein EA, Nonnemaker J, Karns S, Todd JE. Predicting the effects of sugar-sweetened beverage taxes on food and beverage demand in a large demand system. Am J Agric Econ 2014;96(1):1–25. [PubMed](https://www.ncbi.nlm.nih.gov/entrez/query.fcgi?cmd=Retrieve&db=PubMed&list_uids=24839299&dopt=Abstract) <http://dx.doi.org/10.1093/ajae/aat049>

^c^ Zhen C, Wohlgenant M, Karns S, Kaufman P. Habit formation and demand for sugar-sweetened beverages. Am J Agric Econ 2011;93(1):175–93. <http://dx.doi.org/10.1093/ajae/aaq155>

**Table A.2: Maximum Improvement in Profit and Demand Under Alternative Price Elasticity Scenarios For Store B (Selling Sugar-Sweetened Beverages [SSBs] in Cans), Baltimore, Maryland, 2014**

| **Scenario** | **Price Water in $** | **Price SSB in $** | **Percentage Increase Profit** | **Percentage Increase Demand** |
| --- | --- | --- | --- | --- |
| **Lin et al (2011)^a^**  **SSB:** −**0.72**  **Water:** −**0.95** |  |  |  |  |
| Max profit | 1.00 | 0.93 | 4.25 | 0.00 |
| Max demand | 0.87 | 0.93 | 0.07 | 9.33 |
| **Zhen et al (2014)^b^**  **SSB** −**0.73**  **Water** −**0.82** |  |  |  |  |
| Max profit | 1.00 | 0.92 | 6.62 | 0.00 |
| Max demand | 0.80 | 0.93 | 0.41 | 16.40 |
| **Zhen et al (2010)^c^**  **Long-run**  **SSB** −**1.22**  **Water** −**1.10** |  |  |  |  |
| Max profit | 1.00 | 0.88 | 1.81 | 0.00 |
| Max demand | 0.87 | 0.91 | 0.09 | 14.30 |

^a^ Lin B-H, Smith TA, Lee J-Y, Hall KD. Measuring weight outcomes for obesity intervention strategies: the case of a sugar-sweetened beverage tax. Econ Hum Biol 2011;9(4):329–41. [PubMed](https://www.ncbi.nlm.nih.gov/entrez/query.fcgi?cmd=Retrieve&db=PubMed&list_uids=21940223&dopt=Abstract) <http://dx.doi.org/10.1016/j.ehb.2011.08.007>

^b^ Zhen C, Finkelstein EA, Nonnemaker J, Karns S, Todd JE. Predicting the effects of sugar-sweetened beverage taxes on food and beverage demand in a large demand system. Am J Agric Econ 2014;96(1):1–25. [PubMed](https://www.ncbi.nlm.nih.gov/entrez/query.fcgi?cmd=Retrieve&db=PubMed&list_uids=24839299&dopt=Abstract) <http://dx.doi.org/10.1093/ajae/aat049>

^c^ Zhen C, Wohlgenant M, Karns S, Kaufman P. Habit formation and demand for sugar-sweetened beverages. Am J Agric Econ 2011;93(1):175–93. <http://dx.doi.org/10.1093/ajae/aaq155>
